# Effects of Betanin on Pasting, Rheology and Retrogradation Properties of Different Starches

**DOI:** 10.3390/foods11111600

**Published:** 2022-05-29

**Authors:** Taotao Dai, Xiaohong He, Jiahui Xu, Qin Geng, Changhong Li, Jian Sun, Chengmei Liu, Jun Chen, Xuemei He

**Affiliations:** 1 Guangxi Academy of Agricultural Sciences, Nanning 530007, China; ncubamboo@163.com (T.D.); jiansun@gxaas.net (J.S.); 2 Guangxi Key Laboratory of Fruits and Vegetables Storage-Processing Technology, Nanning 530007, China; 3 State Key Laboratory of Food Science and Technology, Nanchang University, Nanchang 330047, China; hexiaohongmaimai@163.com (X.H.); 412314919061@email.ecu.edu.cn (J.X.); 357900210011@email.ecu.edu.cn (Q.G.); lichanghong@163.com (C.L.); liuchengmei@ncu.edu.cn (C.L.)

**Keywords:** betanin, starch, gelatinization, rheology, retrogradation

## Abstract

As a natural pigment with high antioxidative activity, betanin is underutilized owing to less attention. This study aimed to investigate the impact of betanin on pasting, rheology and retrogradation properties of rice, potato and pea starches. Betanin decreased the peak, trough and final viscosity of rice and potato starches, but increased those of pea starch. Rheology measurements implied that betanin had the greatest effect on the hysteresis loops and dynamic modulus of potato starch. Betanin endowed starch pastes with a vivid red appearance and maintained the color of the starch pastes during storage. XRD analysis indicated that betanin weakened the diffraction intensities and reduced the crystallinity of the retrograded starches. Meanwhile, betanin reduced the short-range ordered structure of the retrograde starches. The results of DSC analysis found that betanin significantly depressed the retrogradation enthalpy and retrogradation rate, implying that the long-term retrogradation of starches was delayed. Furthermore, the changed morphology of the retrograded starches was observed. These results suggested that betanin could be applied as an excellent colorant and inhibitor of retrogradation in foods such as bread and pastry products.

## 1. Introduction

Natural pigments have attracted more and more attention owing to the rejection by consumers of synthetic colorants and their adverse effects [1]. Betalains are a type of natural pigments which are commercially obtained from red beet root. In addition, red dragon fruit and pitaya are also abundant sources of betalains, which can be explored as viable alternatives [2]. Betanin is a major betalain and a soluble pigment that is also present in the pitaya fruit [3]. Meanwhile, betanin is a nontoxic betalain approved for use in foods [4] that can be used as a food colorant. In addition, betalains, including betanin, have antioxidant properties [5]. However, previous research about applying betalains mainly concentrated on their stability, due to their sensitivity to thermal and photochemical decomposition. For example, betalains were applied in dairy products, such as cow milk [6] and ice-cream [7], because these products are commonly stored under chilled conditions and could maintain a higher pigment stability. In order to expand the application of this excellent resource, other functions of betalains remain to be explored in addition to being used as a colorant.

Starches serve as an important ingredient, which have been applied in many processed foods such as bakery products, noodles, instant foods and snacks [8]. The physicochemical properties of starches, such as pasting, rheological and retrogradation behaviors, are the main parameters that determine their technological properties and the qualities of their end products. Nevertheless, native starches have some disadvantages, such as weak shear resistance, undesired paste consistency and easy retrogradation. These insufficient characteristics of native starches limited their practical applications in the food industry to a certain extent [9]. Briefly, the retrogradation of starches profoundly affects the textural attributes, shelf life and acceptability of starch-based products [10]. In order to suit specific applications, modification methods, such as chemical, enzymatic and physical methods or some combination of these, were reported to regulate the properties of starches [11]. Recently, plant bioactive substances such as phenolics and plant extracts have been increasingly incorporated to adjust the physicochemical properties of starches. Yu et al. [10] investigated the influences of the selected phenolic acids (cinnamic acid, caffeic acid and ferulic acid) on the retrogradation of corn starch, and found that phenolic acids inhibited the retrogradation of corn starch via the interactions of hydrogen bonding or hydrophobic interaction. Wu et al. [12] suggested that green tea polyphenols reduced pasting attributes, gelatinization enthalpy and the retrogradation degree, as well as improved the freeze–thaw stability of rice starch. Wang et al. [13] reported on the inhibition effect of three common proanthocyanidins (grape seed proanthocyanidins, peanut skin proanthocyanidins and pine bark proanthocyanidins) on the retrogradation properties of maize starch, which was reflected by lowering the melting enthalpy and degree of relative crystallinity. Nevertheless, to our best knowledge, there is little information about the impacts of betanin on the physicochemical properties of starches. Therefore, the aim of this study was to investigate the effects of betanin on pasting, rheology and retrogradation behaviors of rice, potato and pea starches, and the co-gelatinization of betanin and starches was selected as the treatment condition. The results of the study probably provide some useful knowledge for improving the quality of starchy foods, and open up new ideas for the potential utilization of betanin.

## 2. Materials and Methods

### 2.1. Material

Betanin was purchased from Yuanye Bio-Technology Co., Ltd. (Shanghai, China). Rice starch (RS, 10.64% moisture, 0.92% fat, 22.49% amylose, *w*/*w*) was obtained from Sigma-Aldrich Inc. (St. Louis., MO, USA). Potato starch (PoS, 9.78% moisture, fat not detected, 26.54% amylose, *w*/*w*) and pea starch (PeS, 10.47% moisture, 0.49% fat, 38.67% amylose, *w*/*w*) were purchased from Rogate Starch Company (Jiangsu, China). All other chemical reagents were of analytical grade and supplied by Aladdin Chemical Company (Shanghai, China). Distilled water was used throughout the experiments.

### 2.2. Rapid Viscosity Analysis (RVA)

The pasting properties of starches (RS, PoS and PeS) and starch–betanin mixtures were investigated by using an RVA (D8-ADVANCE, Perten, Sweden) according to a previous study [14]. Briefly, the starch sample (2 g) was mixed with betanin at a dosage of 0% or 5% (*w*/*w*) and added to 20 mL of distilled water. The “standard 2” thermal program offered by the supplier was used to determine pasting properties. The samples were held at 50 °C for 60 s, then heated to 95 °C within 225 s and maintained at 95 °C for 150 s, cooled to 50 °C at the same rate as heating, and held at 50 °C for 120 s to develop the final paste viscosity. The rotational speed was set at 960 rpm for the initial 10 s, then changed to 160 rpm. The pasting curves were obtained and parameters were recorded, including peak viscosity (PV), trough viscosity (TV), final viscosity (FV), breakdown (BD) and setback (SB). Some of the gelatinized samples were transferred for rheological and chromaticity value analysis, and others were reserved at 4 °C for 7 days to prepare the retrograded samples. In addition, the same mass ratio of starch to betanin (20:1) was used for DSC analysis of starch–betanin samples.

### 2.3. Rheological Measurements

The rheological characteristics of starches (RS, PoS and PeS) and starch–betanin pastes were analyzed by an MCR 302 rheometer (Anton-Paar, Graz, Austria) with a parallel-plate measuring system, according to He et al. [15]. In brief, the gels of starch and starch–betanin pastes obtained from Section 2.2 were transferred to a rheometer plate with a probe type of PP50 and a gap of 1 mm. The pastes were equilibrated at ambient temperature for 5 min before measurement.

#### 2.3.1. Steady Shear Analysis

The changes in shear stress of the samples were measured within the range of increasing shear rate from 0.01 to 1000 s^−1^ and then decreasing shear rate from 1000 to 0.01 s^−1^ by referencing the method of Zhu et al. [16]. The total area of the hysteresis loops, referring to the region of shuttle between the up and down curve of the fluid properties, was integrated using the Origin software (Version 8.0, Microcal Inc., Northampton, MA, USA). The obtained curve was fitted with the power law model for fitting:

$$\sigma =K\cdot \gamma^{n}$$

where *σ* is the shear stress (Pa), *γ* is the shear rate (s^−1^), *K* is the consistency coefficient (Pa·s^n^), and *n* is the flow behavior index (*n* < 1 for a shear-thinning fluid and *n* = 1 for a Newtonian fluid).

#### 2.3.2. Dynamic Rheological Analysis

Firstly, deformation sweep tests were carried out to determine the maximum deformation attainable by all samples, and the strain γ ranged from 0.01% to 100% at a constant frequency of 1 Hz. The linear viscoelastic region for all samples was in the strain range of 0.1~1.6%. An oscillatory frequency sweep measurement was conducted at room temperature with 1% strain (within the linear viscoelastic (LVE) region of all samples), and a frequency range of 0.1~20.8 Hz was selected according to the previous method [17,18]. The storage modulus (G′) and loss modulus (G″) were obtained, and the loss factor tanδ (G″/G′) was calculated according to the modulus.

### 2.4. The Chromaticity Value Analysis

The chromaticity value of the samples was measured by a colorimeter (CM-5, Ke Sheng Instrument Co., Ltd., Shanghai). Before measurement, the instrument was calibrated with the black and the white board. During measurement, the 10 g sample obtained from Section 2.2 was moved into the sample plate to record the L* value (brightness), a* value (red-green) and b* value (yellowish-blue).

### 2.5. X-ray Diffraction (XRD)

The crystalline properties of the native starches, retrograded starches and starch–betanin samples were determined by a diffractometer (D8 ADVANCEX, BRUKER, Germany) according to a previous study [19]. The samples were scanned from 5 to 40° (2θ) at a scan step size of 0.02° (2θ). The relative crystallinity was calculated as the ratio of the area of the crystal region to total area using the Origin software with the following equation:

$$Relative\;crystallinity\;(\%)=\frac{A_{c}}{A_{c}{+A}_{a}}\;\times 100\;$$

where *A_c_* and *A_a_* represent the crystalline and amorphous areas.

### 2.6. Fourier Transform-Infrared (FT-IR) Spectroscopy

Spectroscopic properties of starches, betanin and starch–betanin samples were characterized by using an FT-IR spectrometer (Thermo Nicolet-5700, Nicolet, Rhinelander, WI, USA) according to the study by Li et al. [20]. The retrograded starch or starch–betanin samples (1–3 mg) were ground with KBr (140 mg) with an agate mortar, and then compressed into disk-shaped pellets. The FT-IR spectra were recorded over the range of 4000 to 400 cm^−1^. Raw spectra were deconvoluted by using Omnic 8.0 software to obtain 1047/1022 cm^−1^ and 995/1022 cm^−1^ values.

### 2.7. Differential Scanning Calorimetry (DSC)

The thermal properties of starches (RS, PoS and PeS) and starch–betanin samples were analyzed by using DSC (7000X, HITACHI, Japan) based on a previous study [21]. Samples (2–3 mg) were accurately weighed and placed in an aluminum pan. Distilled water was added to the pan, and the mass ratio of sample/water was 1:2. The samples were heated from 40 to 100 °C at a rate of 10 °C/min, and the empty pan was used as the reference. The onset (T_o_), peak (T_p_), conclusion (T_c_) temperature and gelatinization enthalpy (Δ*H_g_*) were obtained from the DSC curve. The retrograded samples obtained from Section 2.2 were lyophilized and milled. Then the retrograded starch–betanin samples were reheated in the same conditions to determine the retrogradation enthalpy (Δ*H_r_*). Finally, the degree of retrogradation rate *R* was calculated according to the ratio of Δ*H_r_* and Δ*H_g_*.

### 2.8. Scanning Electron Microscopy (SEM)

Morphological properties of the retrograded starches and starch–betanin samples were observed using SEM (JSM 6701F, JEOL, Japan). The longitudinal section of each freeze-dried sample was fixed on a metal sample holder using double-backed cellophane tape, and then sprayed with a layer of gold to a level of 250–500 nm at an operating voltage of 5 kV. The magnification was 100 times.

### 2.9. Statistical Analyses

The results were expressed as means ± standard deviation (SD) of triplicate analyses for each sample. Data analysis adopted Duncan’s test using SPSS 24.0 statistical software, and differences were considered to be significant at *p* < 0.05.

## 3. Results and Discussion

### 3.1. Pasting Properties of Starches

**Figure 1:**
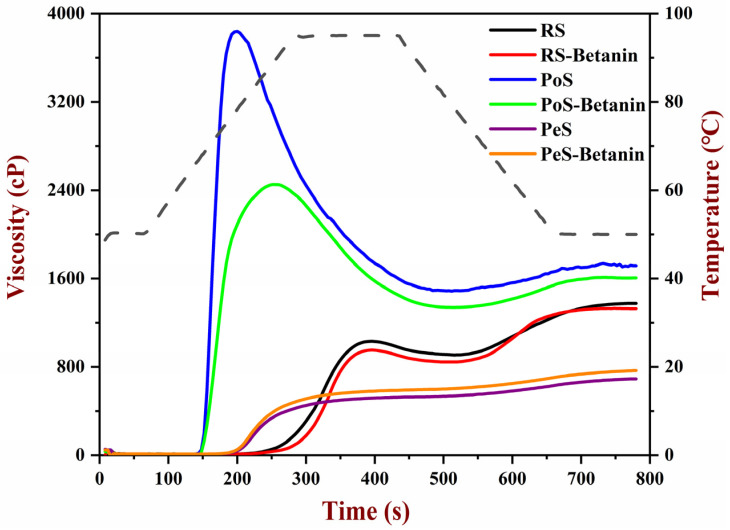
RVA pasting profiles of starches and starch–betanin samples. RS: rice starch; PoS: potato starch; PeS: pea starch.

The pasting properties of different starches and starch–betanin samples were shown in Figure 1 and Table 1. RS and PoS exhibited typical pasting curves with significant convex and concave peaks. Upon heating, the starches swelled early and leached out amylose, which resulted in an increase in viscosity and displaying PV. The PV value of RS and PoS was 1032 and 3839 mPa·s, respectively. When constantly shearing at 95 °C, starch granules were disintegrated, and the BD value represented the degree of disintegration. The BD value of RS and PoS was 124 and 2353 mPa·s, respectively. Subsequently, cooling promoted the rearrangement of amylose molecules, and short-term retrogradation occurred. However, in the pasting patterns of PeS, the concave peak was extremely weak (Figure 1) and the TV value was close to that of the PV, accompanied by a very small BD value. Generally, pea starches were characterized by a high amylose content [22]. As reported by Han et al. [23,24], pasting properties of starches are related to their amylose content. The discrepancy of pasting patterns between RS, PoS and PeS may be affected by their amylose content, resources and other inherent characteristics of starch.

For starch–betanin samples, the combination with betanin affected pasting properties in varying degrees, and the change in pasting parameters for PoS was the biggest. Meanwhile, betanin lowered the PV, TV, BD and FV of RS and PoS. On the contrary, these parameters of PeS were slightly increased. The carboxylate groups in betanin interacted with phosphate groups in PoS, which impeded the absorption of water, swelling of PoS, and leaching out of amylose, thus significantly reducing the viscosity parameters of PoS. In comparison with PeS, RS, with a relatively high content of lipids, was prone to form a complex with betanin; this situation was also not conducive for RS to absorb water and gelatinize. Nevertheless, PeS had a high amylose content and steric hindrance owing to the presence of betanin was beneficial for the interactions between amylose molecules during gelatinization. Despite the increasing or decreasing of the viscosity parameters of starches, the combination with betanin just slightly elevated the SB value of the three starches, indicating the weak ability of betanin to inhibit the short-term retrogradation of starches.

### 3.2. Rheological Properties of Starches

#### 3.2.1. Steady Shear Rheological Properties

**Figure 2:**
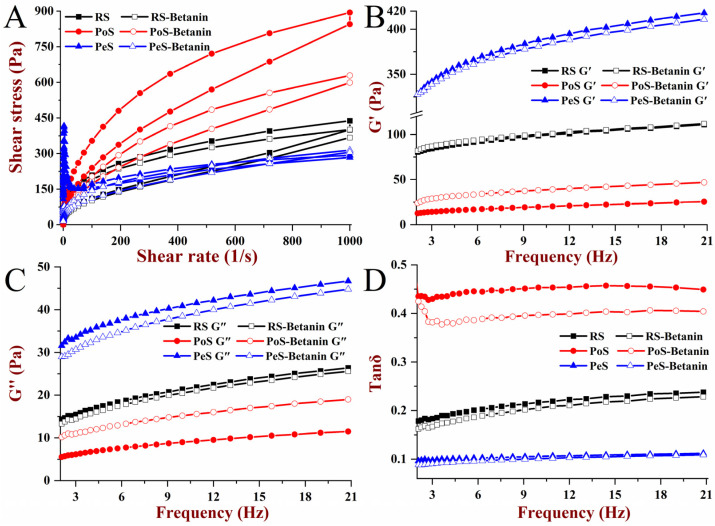
Shear rheology curves (**A**), storage moduli (G′) (**B**), loss moduli (G″) (**C**) and loss tangents (tanδ) (**D**) of starches and starch–betanin samples determined by rheological measurements. RS: rice starch; PoS: potato starch; PeS: pea starch.

The steady shear rheology curves of starch and starch–betanin pastes are shown in Figure 2A. RS and PoS exhibited typical upward and downward curves as a function of shear rate, and hysteresis loops were observed. However, PeS showed an irregular profile of shear stress, and it was difficult to obtain a hysteresis loop. It is likely that PeS pastes easily and rapidly formed a fragile gel during measurement due to their high content of amylose. The shear stress of RS–betanin and PoS–betanin pastes were significantly lower than that of RS and PoS at the shear rate ranges of 0.01~1000 s^−1^ and 1000~0.01 s^−1^; that is, the corresponding apparent viscosity of RS and PoS was lowered when combined with betanin. As for PeS–betanin, typical curves of shear stress and a small hysteresis loop (8943.9 Pa·s^−1^) were present, indicating that betanin improved the shear stability of PeS. Additionally, the hysteresis loop area of RS–betanin (30,553.2 Pa·s^−1^) and PoS–betanin (25,944.7 Pa·s^−1^) was smaller than RS (31,483.2 Pa·s^−1^) and PoS (52,972.6 Pa·s^−1^), respectively (Table 2). Betanin had the biggest effect on the hysteresis loop area of PoS, as the hysteresis loop area was reduced by 40.6%, which indicated that betanin reduced the thixotropy and enhanced the shear stability of starch paste, especially for PoS. The fitting results of the shear stress curves to the power law model are shown in Table 2. Owing to an irregular shear stress curve, PeS was unable to fit the model. Except for PeS, the *R*^2^ of shear stress curves for other samples was greater than 0.97, indicating the high fitting accuracy to the power law model. The fluid indexes *n* of the fitted starches and starch–betanin pastes were all less than 1, indicating that they were typical pseudoplastic fluids with shear thinning behavior. Betanin seemed to have no effect on the *n* of RS and PoS, but reduced their consistency coefficient *K*. It was implied that betanin weakened the thickening property and enhanced the pseudoplasticity of RS and PoS. Meanwhile, betanin endowed PeS with shear thinning behavior, and the biggest *K* and the smallest *n* were presented for PeS–betanin.

#### 3.2.2. Dynamic Rheological Properties

A dynamic frequency sweep range from 0.1 to 20.8 Hz was employed to investigate the viscoelastic properties of starch and starch–betanin pastes, and their storage modulus (G′), loss modulus (G″) and loss tangent (tan δ) as functions of frequency were depicted in Figure 2B–D, respectively. Owing to the unstable state in the measurements of initial small frequency, the results were recorded from 0.6~20.8 Hz. G′ and G″ represent the elasticity and viscosity of the tested samples, and tanδ represents the ratio of G″ and G′, which can be used to explain the viscoelastic behavior [25]. G′ and G″ of all samples were increased along with the frequency, and G′ was bigger than G″, indicating that starch and starch–betanin were typical weak gels. Compared to RS and PoS, PeS had the highest moduli, with the reason possibly being that the high content of amylose contributed to promoting the crosslinking of gel networks. The combination with betanin affected the dynamic rheological properties of starches, and the change in the degree of PoS was the most significant, which was similar to that of the pasting properties. Furthermore, the effect of betanin on G′ was stronger than that of G″; thus, betanin performed a more effective influence on the elasticity than on the viscous properties of RS, PoS and PeS. The tanδ values of RS, PoS and PeS were less than 1 over the whole frequency range, and PeS exhibited the lowest tanδ values, showing the strongest elastic behavior. The addition of betanin decreased the tanδ value of starches; that is, starch–betanin exhibited lower tanδ values than that of the corresponding starch. The effect of betanin on the tanδ of PoS was the biggest, and the smallest effect was observed on the tanδ value of PeS. The decreased tanδ values implied that the structure of the gel network was enhanced, which was probably attributed to increasing the junctions or crosslinking between amylose and swollen fragments caused by the presence of betanin. The result demonstrated that betanin changed the network structure of PoS pastes to a greater extent compared to RS and PeS, which was consistent with the result of the steady shear rheological properties.

### 3.3. Color Observation

**Figure 3:**
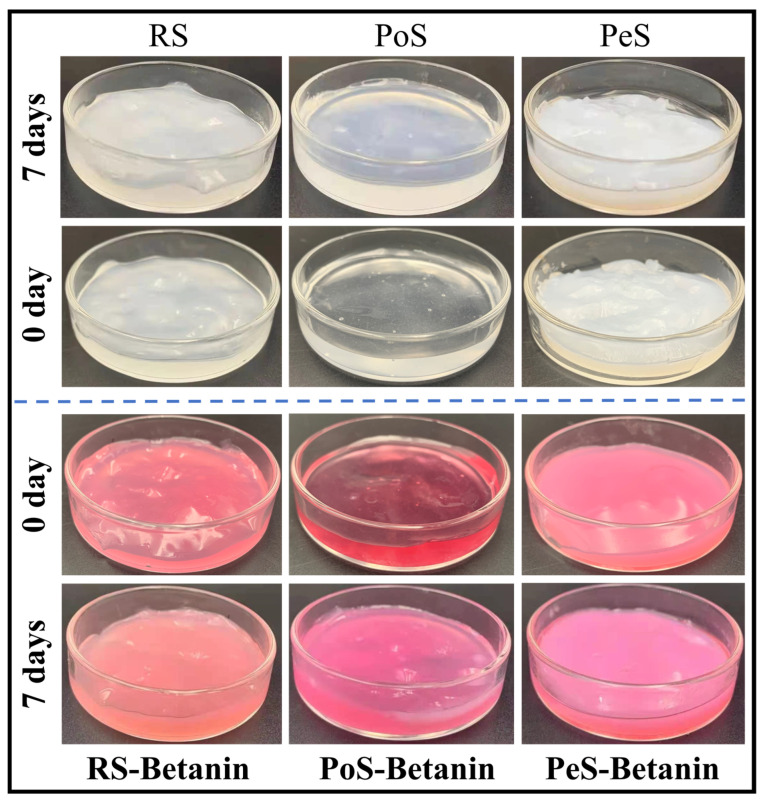
The appearance of starches and starch–betanin samples after retrogradation for 0 days and 7 days. RS: rice starch; PoS: potato starch; PeS: pea starch.

In addition to its high antioxidant activity and many health-beneficial effects, betanin is a natural food colorant [26,27]. Therefore, observing the changes in the color of pastes could help foresee the visual quality of pastes during storage, which plays an important role in determining the acceptability and desirability of the products for consumers. Figure 3 displays the appearance of starch and starch–betanin pastes during retrogradation, and the lightness (L*), redness (a*) and yellowness (b*) of these samples are listed in Table 3. The fresh PoS paste was transparent, and RS and PeS pastes tended to be white. The L* value for PoS (29.67) was the lowest, and those of RS and PeS pastes were 40.17 and 57.85, respectively. Nevertheless, fresh starch–betanin pastes exhibited a vivid and attractive red color, which indicated that betanin could endow starch paste with a good visual image. As shown in Table 3, the values of a* for fresh starch–betanin pastes were also significantly higher than that of the corresponding fresh starch pastes. After retrogradation for 7 days, L* values of pure starch pastes were higher than that of the corresponding fresh starch paste. Starch molecules were rearranged to form crosslinking during the retrogradation, resulting in the color to gradually become cloudy. The transparency was decreased and the whiteness was increased, giving rise to the increasing of lightness. In regard to the starch–betanin pastes, similar alterations as occurred to the pure starch pastes after retrogradation were found. Starch–betanin pastes after retrogradation for 7 days presented higher L* and a* values than fresh starch–betanin pastes. In light of the analysis of retrogradation properties in the next sections, betanin inhibited the long-term retrogradation of all starches. In other words, betanin delayed the transformation to cloudy; thus, the redness a* of starch–betanin pastes was increased after storage. According to the appearance of the pastes, the retrograded starch–betanin pastes seemed to slightly fade, which was mainly related to the phenomenon of becoming cloudy during retrogradation. Overall, the retrograded starch–betanin pastes maintained a bright red color, which indicates that betanin can also be applied as a good colorant during the storage of starches.

### 3.4. XRD Analysis

**Figure 4:**
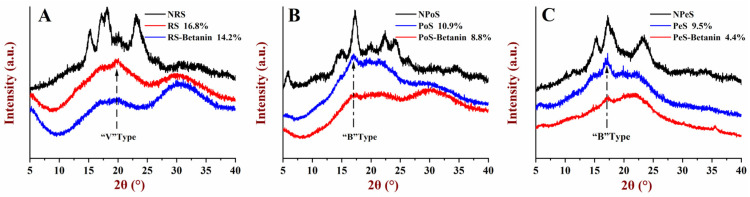
XRD diagrams of starches and starch–betanin samples after retrogradation; (**A**) rice starch, (**B**) potato starch, and (**C**) pea starch. NRS: native rice starch; NPoS: native potato starch; NPeS: native pea starch. RS: retrograded rice starch; PoS: retrograded potato starch; PeS: retrograded pea starch.

The X-ray diffraction patterns of the retrograded starch and starch–betanin samples were displayed in Figure 4. Native rice starch (NRS), native potato starch (NPoS) and native pea starch (NPeS) exhibited typical A-, B- and C-type diffraction patterns, respectively. After retrogradation for 7 days, RS with an A-type diffraction pattern was transformed to a V-type pattern, and B-type patterns for the retrograded PoS and PeS were shown. The relative crystallinity of the retrograded RS, PoS and PeS was 16.8%, 10.9% and 9.5%. The lowest crystallinity of the retrograded PeS may be related to its low amylopectin content, because the crystallinity of the processed starch was considered to be associated with retrogradation of amylopectin [28]. In regard to all the retrograded starch–betanin samples, their diffraction intensities were observably weakened in comparison with the retrograded starch. For example, the diffraction peak at 19.6° for the retrograded RS–betanin almost disappeared, and peaks at 17.3° for the retrograded PoS–betanin and PeS–betanin narrowed significantly. The relative crystallinity was reduced by 14.2%, 8.8% and 4.4% for the retrograded RS–betanin, PoS–betanin and PeS–betanin, respectively. These results imply that betanin impeded the rearrangement of starch molecules during long-term retrogradation. The presence of betanin may alter the distribution and rearrangement of starch molecules, and retrogradation was restrained by steric hindrance. The degree of inhibiting crystallinity by betanin was the highest for PeS, and it is likely that betanin and the high amylose content of PeS occupied the space to interfere with the association of amylopectin.

### 3.5. Short-Range Ordered Structure Analysis

**Figure 5:**
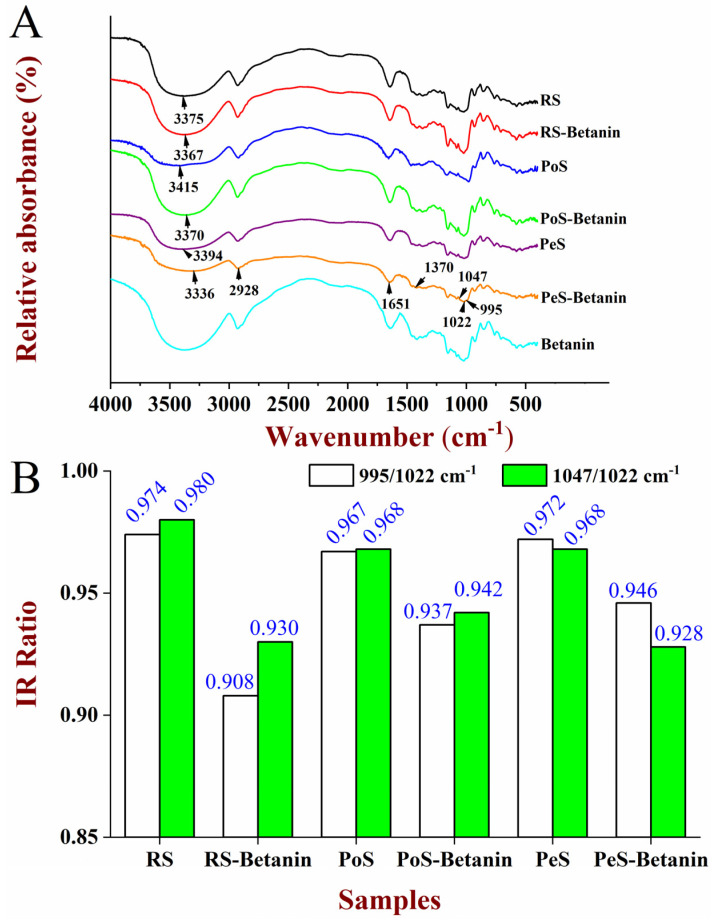
FTIR spectra (**A**) and 1047 cm^−1^/1022 cm^−1^ ratios from deconvoluted FTIR spectra (**B**) of starches and starch–betanin samples after retrogradation for 7 days. RS: rice starch; PoS: potato starch; PeS: pea starch.

Internal structural changes of starches after retrogradation could also be determined by a FTIR spectrometer that was used to analyze the short-range ordered structure, and the FITR spectra of starch and starch–betanin after retrogradation for 7 days were displayed in Figure 5A. Compared to the retrograded starches, no new characteristic absorption peaks appeared in the spectra of the retrograded starch–betanin samples, and just some of the wavenumbers of peaks were shifted, which implied that the primary structure of the retrograded starches was maintained and no covalent bond was formed. As demonstrated by Sevenou et al. [29], the absorbance at approximately 995 cm^−1^, 1047 cm^−1^ and 1022 cm^−1^ could be associated with the structural order of starch chains near the granule surface. Meanwhile, the ratio of 1047/1022 cm^−1^ and 995/1022 cm^−1^ could be used to determine a degree of short-range order for the retrograded starches [30]. As displayed in Figure 5B, the retrograded starch–betanin behaved with lower ratio values of 1047/1022 cm^−1^ and 995/1022 cm^−1^ as compared to the retrograded starch. For example, the ratios of 995/1022 cm^−1^ for RS, PoS and PeS were 0.974, 0.967 and 0.972, respectively. However, the ratios of RS–betanin, PoS–betanin and PeS–betanin were decreased to 0.908, 0.937 and 0.946, respectively. These results suggest that betanin reduced the short-range order structure of the retrograded starches, which was in accord with the effect of betanin on crystallinity in the XRD analysis.

### 3.6. Thermal Properties Analysis

The thermal and retrogradation properties of starch and starch–betanin determined by DSC are presented in Table 4. The presence of betanin increased the gelatinization transition temperatures (T_o_, T_p_ and T_c_) of all starches, and it was implied that starch gelatinization was delayed by betanin, which might be related to the strong hydrophilicity of betanin and its ability to hinder starch from absorbing water. Δ*H_g_* represents the required thermal energy for melting the double-helix structure and destroying crystallinity within the starch granule [31]. In comparison to pure starches, a lower Δ*H_g_* for starch–betanin was found. Similar results were reported in our previous paper [14], in which the addition of polymeric proanthocyanidin decreased the gelatinization enthalpy of RS, PoS and PeS. Simultaneously, betanin significantly delayed the long-term retrogradation of the three starches, resulting in a reduction in the retrogradation enthalpy (Δ*H_r_*) and retrogradation rate (*R*). After incorporating betanin, the *R* of RS, PoS and PeS declined from 34.13%, 42.95% and 31.82% to 23.24%, 31.48% and 25.80, respectively. It was indicated that betanin affected the formation and weakened the order degree of crystallinity during the long-term retrogradation of starches, which conformed to the results of the XRD and FITR analyses. In light of these results, it is known that betanin could be used as an excellent inhibitor of retrogradation in products such as bread and pastry.

### 3.7. Morphology Analysis

**Figure 6:**
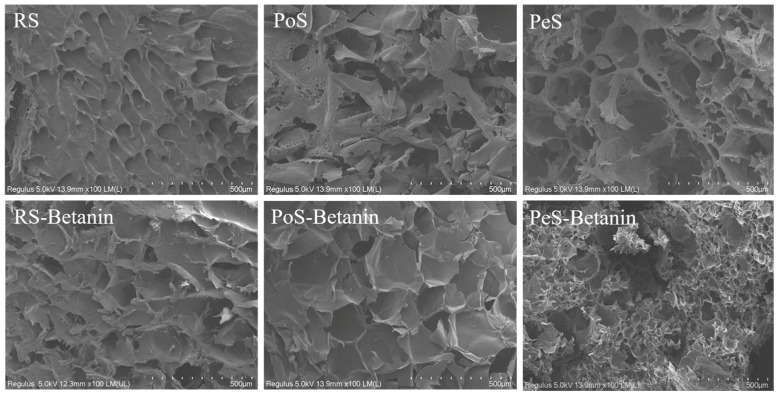
SEM photographs of starches and starch–betanin samples after retrogradation for 7 days. Magnification was 100. RS: rice starch; PoS: potato starch; PeS: pea starch.

Microstructures of the retrograded starches and starch–betanin samples were examined through SEM to observe the changes from adding betanin, and images are depicted in Figure 6. The retrograded RS displayed a dense surface with a uniformly distributed cavity, and the retrograded PoS and PeS presented relatively rough network-like structures with some fragments. Nevertheless, the incorporation of betanin altered the formation of the gel network of the retrograded starches, and the retrograded starch–betanin samples displayed more porous and loose structures. In comparison with the morphology of the retrograded starches, RS–betanin and PoS–betanin showed an increased cavity volume, and porous spongy-like morphology was observed for PeS–betanin. This phenomenon was similar to the report by Xu et al. [32], who found *Vaccinium bracteatum Thunb. leaf* pigment loosened matrices of rice starch gels. The microstructural changes reflected that the addition of betanin affected the gel network of the retrograded starches. That is, betanin might inhibit the long-term retrogradation of starches, which coincides with the results of the aforementioned investigations.

## 4. Conclusions

The pasting, rheology and retrogradation properties of rice, potato and pea starches were changed by the presence of betanin. Betanin decreased the peak, trough and final viscosity of rice and potato starches, but increased these of pea starch. Rheological properties including thixotropy, the dynamic modulus and the loss factor of the three starches were varied by different degrees after incorporating betanin. Betanin endowed starch pastes with a vivid red appearance and maintained color during storage. Furthermore, the poor short-range molecular order, low crystallinity and low retrogradation enthalpy of starches were induced by betanin during retrogradation, suggesting that betanin could inhibit the retrogradation of starches. The micromorphology of the retrograded starches was also altered by betanin. These findings provide guidance for the application of betanin in developing foods that require colorant and an inhibitor of starch retrogradation.

## Figures and Tables

**Table 1:** Pasting properties of starches and starch–betanin samples.

Samples	Peak Viscosity (mPa·s)	Trough Viscosity (mPa·s)	Breakdown (mPa·s)	Final Viscosity (mPa·s)	Setback (mPa·s)
RS	1032 ± 4 ^c^	908 ± 8 ^c^	124 ± 7 ^c^	1375 ± 8 ^c^	467 ± 1 ^b^
RS–betanin	953 ± 8 ^d^	845 ± 6 ^d^	108 ± 10 ^d^	1328 ± 6 ^d^	483 ± 5 ^a^
PoS	3839 ± 18 ^a^	1486 ± 21 ^a^	2353 ± 10 ^a^	1716 ± 15 ^a^	230 ± 12 ^d^
PoS–betanin	2490 ± 11 ^b^	1339 ± 2 ^b^	1151 ± 6 ^b^	1611 ± 9 ^b^	272 ± 9 ^c^
PeS	522 ± 8 ^f^	506 ± 6 ^f^	16 ± 6 ^e^	692 ± 6 ^f^	186 ± 5 ^f^
PeS–betanin	586 ± 6 ^e^	568 ± 2 ^e^	18 ± 4 ^e^	767 ± 3 ^e^	199 ± 5 ^e^

Differences between values, indicated by different letters in the same columns, are significant at 0.05 level of confidence. RS: rice starch; PoS: potato starch; PeS: pea starch.

**Table 2:** The steady shear rheological parameters of starches and starch–betanin samples.

Samples	Hysteresis Loop Area (Pa·s^−1^)	Up Curve			Down Curve		
		*K/*Pa·s^n^	*n*	*R^2^*	*K/*Pa·s^n^	*n*	*R^2^*
RS	31483.2	54.67	0.30	0.98	12.98	0.48	0.98
RS–betanin	30553.2	53.98	0.28	0.99	11.84	0.48	0.98
PoS	52972.6	52.76	0.50	0.98	22.63	0.52	0.98
PoS–betanin	25944.7	21.01	0.41	0.97	16.06	0.52	0.98
PeS	-	-	-	-	-	-	-
PeS–betanin	8943.9	56.01	0.23	0.97	24.92	0.35	0.97

RS: rice starch; PoS: potato starch; PeS: pea starch; *K*: the consistency coefficient; *n*: the flow behavior index.

**Table 3:** The color of starches and starch–betanin samples after retrogradation for 0 days and 7 days.

Samples	Storage Time	L*	a*	b*
RS	0 days	40.17 ± 0.02 ^g^	−0.96 ± 0.00 ^i^	−8.57 ± 0.01 ^h^
RS	7 days	48.73 ± 0.02 ^d^	−1.74 ± 0.00 ^j^	−7.81 ± 0.02 ^f^
RS–betanin	0 days	35.30 ± 0.01 ^j^	9.30 ± 0.04 ^e^	−5.55 ± 0.01 ^d^
RS–betanin	7 days	41.77 ± 0.01 ^f^	10.26 ± 0.04 ^d^	−2.52 ± 0.02 ^c^
PoS	0 days	29.67 ± 0.02 ^k^	−0.40 ± 0.01 ^g^	−1.39 ± 0.00 ^a^
PoS	7 days	37.18 ± 0.01 ^h^	−0.64 ± 0.01 ^h^	−8.07 ± 0.01 ^g^
PoS–betanin	0 days	27.41 ± 0.07 ^l^	3.96 ± 0.03 ^f^	−1.49 ± 0.01 ^b^
PoS–betanin	7 days	35.56 ± 0.01 ^i^	10.69 ± 0.01 ^c^	−8.69 ± 0.01 ^i^
PeS	0 days	57.85 ± 0.02 ^b^	−2.19 ± 0.00 ^k^	−9.49 ± 0.01 ^k^
PeS	7 days	66.03 ± 0.02 ^a^	−2.57 ± 0.00 ^l^	−8.96 ± 0.01 ^j^
PeS–betanin	0 days	41.97 ± 0.01 ^e^	16.36 ± 0.03 ^b^	−7.58 ± 0.01 ^e^
PeS–betanin	7 days	50.77 ± 0.00 ^c^	20.92 ± 0.01 ^a^	−10.54 ± 0.01 ^l^

Differences between values, indicated by different letters in the same columns, are significant at 0.05 level of confidence. RS: rice starch; PoS: potato starch; PeS: pea starch; L*: brightness value; a*: red-green value; b*: yellowish-blue value.

**Table 4:** Thermal properties of starches and starch–betanin samples after retrogradation for 7 days.

Samples	T_o_ (°C)	T_p_ (°C)	T_c_ (°C)	Δ*H_g_* (J/g)	Δ*H_r_* (J/g)	*R* (%)
RS	63.65 ± 0.10 ^c^	66.16 ± 0.03 ^c^	70.54 ± 0.47 ^c^	8.41 ± 0.39 ^d^	2.87 ± 0.12 ^c^	34.13
RS–betanin	64.76 ± 0.11 ^b^	68.00 ± 0.16 ^b^	73.49 ± 0.82 ^a^	6.11 ± 0.30 ^e^	1.42 ± 0.11 ^d^	23.24
PoS	58.91 ± 0.41 ^e^	63.23 ± 0.21 ^e^	66.54 ± 0.26 ^d^	12.69 ± 0.41 ^a^	5.45 ± 0.03 ^a^	42.95
PoS–betanin	60.75 ± 0.07 ^d^	65.93 ± 0.51 ^d^	69.74 ± 0.98 ^c^	9.69 ± 0.21 ^c^	3.05 ± 0.07 ^c^	31.48
PeS	60.44 ± 0.35 ^d^	67.73 ± 0.38 ^b^	71.87 ± 0.24 ^b^	12.41 ± 0.13 ^a^	3.95 ± 0.26 ^b^	31.82
PeS–betanin	70.82 ± 0.26 ^a^	71.80 ± 0.41 ^a^	74.21 ± 0.13 ^a^	11.55 ± 0.34 ^b^	2.98 ± 0.03 ^c^	25.80

Differences between values, indicated by different letters in the same columns, are significant at 0.05 level of confidence. RS: rice starch; PoS: potato starch; PeS: pea starch. T_o_: onset temperature; T_p_: peak temperature; T_c_: conclusion temperature; Δ*H_g_*: gelatinization enthalpy; Δ*H_r_*: retrogradation enthalpy; *R*: the degree of retrogradation (Δ*H_r_*/Δ*H_g_*) * 100.

## Data Availability

The data presented in this study are available in this article.
